# Anticancer Effects of Gut Microbiota-Derived Short-Chain Fatty Acids in Cancers

**DOI:** 10.4014/jmb.2301.01031

**Published:** 2023-03-30

**Authors:** Mi-Young Son, Hyun-Soo Cho

**Affiliations:** 1 Korea Research Institute of Bioscience and Biotechnology, Daejeon 34141, Republic of Korea; 2 Korea University of Science and Technology, Daejeon 34113, Republic of Korea; 3 Department of Biological Science, Sungkyunkwan University, Suwon 16419, Republic of Korea

**Keywords:** Cancer, gut microbiota, short-chain fatty acids, butyrate, combination therapy

## Abstract

Short-chain fatty acids (SCFAs), such as butyrate, propionate, and acetate produced by the gut microbiota have been implicated in physiological responses (defense mechanisms, immune responses, and cell metabolism) in the human body. In several types of cancers, SCFAs, especially butyrate, suppress tumor growth and cancer cell metastasis via the regulation of the cell cycle, autophagy, cancer-related signaling pathways, and cancer cell metabolism. In addition, combination treatment with SCFAs and anticancer drugs exhibits synergistic effects, increasing anticancer treatment efficiency and attenuating anticancer drug resistance. Therefore, in this review, we point out the importance of SCFAs and the mechanisms underlying their effects in cancer treatment and suggest using SCFA-producing microbes and SCFAs to increase therapeutic efficacy in several types of cancers.

## Introduction

Cancer mortality is the highest among all disease-related deaths worldwide. Cancer cells proliferate uncontrollably, and they reproduce and move freely (via migration and invasion) to other parts of the body. And cancer types such as carcinoma, leukemia, lymphoma, and sarcoma are classified on the basis of their tissue of origin [1]. For cancer treatment, chemotherapy (5-fluorouracil (5-FU), tamoxifen, oxaliplatin and paclitaxel, etc.) is targeted to fast-growing cancer cells to inhibit DNA synthesis or arrest the cell cycle [2-4]. However, several side effects (hair loss, anemia, bleeding, infections, tiredness, etc.) are common in cancer patients who receive chemotherapy. To overcome and reduce side effects and increase drug therapy efficacy, target-specific inhibitors/antibodies or immunotherapies (programmed cell death 1 (PD-1) and programmed cell death ligand 1 (PD-L1)) have been used to treat several types of cancers [5-8]; however, more effective and safer anticancer drugs and supplemental alternatives are continuously needed.

Recently, several papers reported that the composition of the gut microbiota is markedly changed in cancer patients compared with that in normal healthy people [9] and demonstrated that metabolites derived from the gut microbiota suppress the growth and metastasis of cancer cells by regulating cancer-specific signaling pathways or oncogene/tumor- suppressor gene expression [10]. Among these metabolites, short-chain fatty acids (SCFAs; butyrate, propionate, and acetate) are the main metabolites produced via bacterial fermentation of dietary fibers and are related to crucial physiological effects [11], including immune responses [12], defense mechanisms [13], and cell metabolism [14] in the human body. Moreover, SCFAs, mainly butyrate, effectively suppress cell growth, migration, and invasion of cancer cells via the inhibition of histone deacetylase (HDAC) activity [15] and the regulation of cancer-related gene expression, showing synergistic effects with anticancer drugs such as 5-FU [16] and oxaliplatin [17]. Additionally, SCFA-producing microbes changed the composition of the gut microbiota and suppressed cancer growth and metastasis in in vivo models [18].

In this review, we outline the current knowledge about cancer cell regulation, particularly via suppression mechanisms, induced by SCFAs in several types of cancer. We also highlight the roles of SCFA-producing microbes in cancer treatment. In addition, we describe the advantages and synergistic effects of SCFAs and SCFA-producing microbes for use with anticancer drug treatment to increase therapeutic efficacy and present the possibility of alternative cancer treatments.

## Colorectal Cancer (CRC)

CRC is the 3^rd^ most common cancer in the world and is associated with several signs and symptoms, such as rectal bleeding, leading to blood in stool. For CRC treatment, chemotherapy such as 5-FU or targeted therapy (cetuximab) is widely used [19]; however, several side effects of cancer therapy and low therapeutic efficacy have been recognized as obstacles to effective CRC treatment. Recently, butyrate-producing gut microbes have received attention for use in CRC treatment. After treatment with culture supernatant, including butyrate from *Lactobacillus plantarum* strains, HT-29 cell line proliferation was suppressed via the downregulation of cyclin D1 expression, which led to arrest in the G2/M phase of the cell cycle [20]. Additionally, butyrate-producing *Butyricicoccus pullicaecorum* fed to CRC-bearing mice led to an increase in body weight and a reduction in serum carcinoembryonic antigen levels. The expression of SCFA transporters such as solute carrier family 5 member 8 (SLC5A8) and G protein-coupled receptor 43 (GPR43) was increased by treatment with sodium butyrate (NaB) in SW480 and SW620 CRC cell lines [21]. Chan *et al* demonstrated that *B. pullicaecorum* also suppressed the proliferation of colon cancer cells via downregulation of chromosome segregation 1 like (CSE1L) expression [22]. *Clostridium butyricum* is also a butyrate-producing bacterium. *C. butyricum* inhibited high-fat diet (HFD)-induced intestinal tumor development by decreasing the levels of pathogenic bacteria and bile acid-biotransforming bacteria and increasing the level of SCFA-producing bacteria in an Apc^min/+^ mouse model. Moreover, the culture supernatant of *C. butyricum* and NaB induced cell apoptosis by suppressing Wnt/beta-catenin and increased the expression levels of GPR43 and GPR109A in the HCT 116 cell line, as determined via an analysis of GPR 43 and GPR 109A expression in CRC tissue compared to that in normal colonic tissue [23]. In addition, the propionate-producing bacterium *Bacteroides thetaiotaomicron* produced an effect similar to that of NaB treatment in CRC cell lines. Treatment with *B. thetaiotaomicron* culture supernatant and sodium propionate (NaP) clearly suppressed the proliferation of CRC cell lines and increased the cell apoptosis rate [19].

Via epigenetic modification regulation, the microRNA (miRNA) expression of miR-181a, miR-125b, miR-593, and miR-1227 expression was significantly increased by butyrate treatment in colon cancer cell lines, as determined with a high-throughput screening system. These miRNAs were clearly involved in cell cycle arrest and the repression of cell growth by regulating the WNT and PI3K signaling pathways. Combination treatment with miRNA mimics and NaB in colon cancer cell lines induced a synergistic effect that repressed cell growth and regulated the expression of cancer-related genes [24]. NaB also regulates the TCA cycle and methionine metabolism via its effect on epigenetic machinery. Specifically, NaB critically regulates epigenetic modifiers such as DNA methyltransferase 1 (DNMT1), lysine demethylase 1A (KDM1A) and 1B, histone acetyltransferase 1 (HAT1), and tet methylcytosine dioxygenase (TET1) [25]. Moreover, NaP treatment upregulated HECT domain E3 ubiquitin protein ligase 2 (HECTD2) expression by increasing the histone 3 lysine 27 acetylation (H3K27 ac) rate, leading to proteasomal degradation of euchromatic histone-lysine N-methyltransferase 2 (EHMT2) to suppress the proliferation of CRC cell lines. Finally, cotreatment with NaP and an EHMT2-specific inhibitor (BIX-01294) showed a synergistic effect on cell apoptosis in a HCT 116 spheroid model, implying that SCFA-related epigenetic regulation reduced the effect on colon cancer cell proliferation and metastasis [19].

Next, via the molecular mechanisms inhibiting colon cancer progression and metastasis mediated by gut microbiota-derived SCFAs, NaB treatment enhanced the efficiency of radiotherapy in CRC patient-derived organoid models. Compared with the nontreatment group, NaB significantly increased radiation-induced cell death by regulating forkhead box O3A (FOXO3A) gene expression [26]. Glycolysis inhibitors critically regulate glucose metabolism to suppress cancer cell proliferation. NaB treatment decreased the expression of glucose transporter 1 (GLUT1) and glucose-6-phosphate dehydrogenase (G6PD) in HCT 116 cell lines. Additionally, the combination of 5-FU and NaB enhanced 5-FU-related cell apoptosis [27]. Treatment with deoxycholic acid (DCA) induced apoptosis in HCT116 cells. Although DCA-resistant HCT 116 (DCA-RCL) showed a DCA-resistant effect compared with wild-type HCT 116, NaB treatment induced the cell apoptosis of DCA-RCL cells via the downregulation of the cellular myelocytomatosis oncogene (c-Myc) and phosphorylation of p38 in DCA-RCL [28]. Moreover, NaB treatment affected cellular phosphotransfer networks via the regulation of oxidative phosphorylation in the Caco-2 cell line. Hence, the regulation of NaB-related metabolism in CRC cells may become a therapeutic strategy for CRC [29].

In the immune system, the gut microbiota regulates the activation of immune cells and inflammation by controlling innate and adaptive immune signaling pathways, such as the nuclear factor kappa B (NF-κB) pathway [30]. Butyrate and propionate have also been associated with anti-inflammatory effects via their regulation of immune cell migration, adhesion, and cytokine secretion [31, 32]. For example, NaB modulates the immune response through the regulation of natural killer T cells and T helper 17 (Th17) cells to suppress colon-to-liver metastasis [33]. Additionally, propionate induces cell surface expression of the killer cell lectin-like receptor k1 (KLRK1) ligand and MHC class I polypeptide-related sequence A/B (MICA/B) to enhance the immune response in CRC [32]. However, Coutzac *et al* suggested that the systemic concentration of butyrate or propionate in blood prohibited the antitumor activity of anti-CTLA-4 therapy, such as ipilimumab therapy [31].

**Fig. 1:**
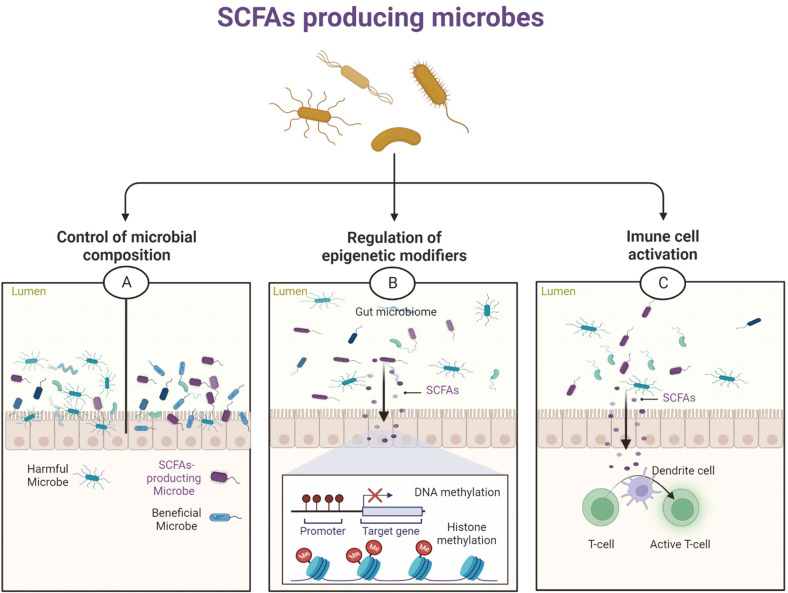
The suppression mechanism by SCFA-producing microbes in CRC. (**A**) SCFA-producing microbes inhibited the levels of pathogenic bacteria and increased the levels of SCFA-producing bacteria and beneficial bacteria. (**B**) Gut microbiota-derived SCFAs regulate epigenetic modifiers for the regulation of chromatin structure. (**C**) Gut microbiota-derived SCFAs associated with the activation of immune cells and inflammation.

Hence, the intake of SCFA-producing microbes or SCFA treatment before or during treatment with anticancer drugs in CRC patients may induce therapeutic efficacy of anticancer drugs by controlling of epigenetic modification regulation, immune cell activation, and gene expression regulation (Fig. 1). However, further studies on the control of SCFA levels for the CRC treatment, such as immunotherapy, are needed.

## Cervical Cancer (CC)

CC develops in the cells of the cervix, and human papillomavirus (HPV) is recognized as an important cause of cervical cancer [34]. The composition of the cervical microbiome was compared between CC patients and healthy premenopausal women; the healthy women mainly presented with a *Lactobacillus* population (more than 90%), but CC patients presented with a low population of *Lactobacillus* and an increase in bacterial genus diversity [35]. For CC treatment, chemotherapy, such as cisplatin and paclitaxel, has been regularly used. To increase the therapeutic efficacy of chemotherapy in CC, a cotreatment consisting of NaB and cisplatin suppressed cell migration and invasion via the downregulation of matrix metallopeptidase 2 (MMP2), MMP7 and MMP9, regulation of the epithelial-mesenchymal transition (EMT) and reduced proliferation of HeLa and Siha cell lines. Additionally, in a mouse subcutaneous xenograft model, although single treatment with NaB or cisplatin presented tumor suppression, cotreatment with NaB and cisplatin showed more synergistic suppression of tumor volume than single treatments [36]. Additionally, combination treatment with NaB and 7-hydroxy-staurosporine (UCN-01) induced cell apoptosis. The induction of p53 and p73 expression by cotreatment with NaB and UNC-01 regulates downstream targets such as BCL2-associated X (BAX), p21, and BCL-2 to induce apoptosis [37]. Ogawa *et al*. reported that free fatty acid receptor 2 (FFAR2; GPR43), an SCFA receptor, was overexpressed in CC, and cotreatment with GLPG (FFAR2 antagonist) and SCFAs attenuated cell growth suppression in HeLa cell lines compared to the effect of only SCFAs [38]. Moreover, NaP treatment increased the apoptosis rate and induced reactive oxygen species (ROS) production and autophagy by regulating the mitochondrial membrane potential and AKT/mTOR signaling, and NaP treatment induced a sub-G1 cell population in the HeLa cell line [39]. Thus, in CC treatment, SCFAs or SCFA-producing bacteria may become alternative methods for increasing the effect of anticancer drugs.

## Gastric Cancer (GC)

GC tumors grow uncontrollably in the stomach and have been reported to be the leading cause of cancer death. *Helicobacter pylori* infection is an important cause of GC, with *H. pylori* infection changing the acidity of the stomach to alter the gastric microbiome [40] and inducing an inflammatory response that breaks the gastric barrier [41]. In the suppression of *H. pylori* growth, NaB treatment clearly showed the bactericidal effect of *H. pylori*. Additionally, NaB treatment decreased the expression of CagA and VacA, and in *H. pylori*-infected mice, NaB uptake also reduced virulence factor production and Toll-like receptor (TLR) expression. Although *H. pylori* infection in mice increased the diversity of the gut microbiota, NaB intake in mice reduced the diversity and abundance of the gut microbiota. Thus, NaB treatment could affect the development of *H. pylori*-induced diseases [42, 43].

To increase the therapeutic efficacy of GC treatment using microbial metabolites, a study of a combination treatment with NaB and cisplatin was performed, and the results showed an increased apoptosis rate in GC cell lines and an in vivo xenograft tumor model by mediating the upregulation of mitochondrion-related apoptosis proteins. Cell migration and invasion were decreased by this combination treatment through downregulation of MMP2 and MMP9 expression [44]. In the gastric mucosal repair process, NaB decreased proinflammatory cytokine levels, attenuated oxidative stress, and induced the expression of mucosal repair factors mediated through the GPR109A receptor in a gastric ulcer mouse model [45]. Among the biomarkers used for GC diagnosis, the levels of SCFAs (propionate and butyrate) and tricarboxylic acid (TCA) cycle intermediates (cis-aconitate, alpha-ketoglutarate, and fumarate) in plasma were significantly decreased in the GC group, implying that the ratio of metabolites might be used as a diagnostic marker in GC [46]. Moreover, a small RNA-seq analysis of GC cell lines treated with butyrate showed that 323 miRNAs were upregulated by butyrate treatment; among these miRNAs, 3 miRNAs (miR-125a-3p, miR-181a-2-3p, and miR-1304-3p) were associated with a poor prognosis for GC [47]. Thus, to increase the therapeutic efficacy of chemotherapy, combination therapy with SCFAs may be an alternative method for GC treatment.

## Lung Cancer (LC)

LC is one of the leading causes of cancer death and is mainly classified into two types: non-small cell lung carcinoma (NSCLC) and small cell lung cancer (SCLC) [48]. In the composition of the microbiome in LC patients, qRT‒PCR analysis specifically showed that the levels of butyrate-producing microbes, such as *Faecalibacterium prausnitzii*, *Clostridium leptum*, *Ruminococcus* spp. were significantly decreased in NSCLC patients compared with healthy adults, as determined with fresh stool samples, suggesting a high correlation between LC and gut microbiota [49]. As revealed through miRNA profiling in the A549 cell line, the expression of miR-3935, miR-574-3p and miR-494-3p was upregulated by NaB treatment. Overexpressed miR-3935 reduced cell migration and proliferation in the A549 cell line through direct regulation of ring finger protein 115 (RNF115) [50]. Additionally, the expression of thioredoxin-interacting protein (TXNIP) was significantly increased by NaB treatment to induce apoptosis and caspase 3/7 activity in the A549 cell line [51]. In conjunction with radiation therapy, NaB treatment of LC induced antiradiation toxicity and changed the expression of 5 core targets (AKT serine/threonine kinase 1 (AKT1), tumor protein P53 (TP53), Sirtuin 1 (SIRT1), Notch receptor 1 (NOTCH), and phosphatase and tensin homolog (PTEN)), protecting cells against radiation-induced lung injury [52]. In immune response, the mice fed VSL#3 probiotics exhibited increased concentrations of propionate and butyrate in the blood and gut. These high SCFA concentrations led to Th17 cell recruitment by inducing C-C motif chemokine ligand 20 (CCL20) expression in the lung; subsequently, Th17 cells inhibited tumor proliferation and metastasis [53]. Propionate treatment induced G2/M phase arrest via the regulation of cell cycle protein Survivin and p21 expression in H1299 and H1703 lung cancer cell lines [54].

In systemic chemotherapy of lung cancer, paclitaxel (PTX) leads to side effects. In an in vivo study, NaB treatment before PTX treatment restored the microbiota composition, food intake levels, and gut barrier integrity and thus attenuated PTX-induced depression [55]. In addition, cotreatment with NaB and docetaxel led to a higher apoptosis rate than either single treatment in lung cancer cell lines. This combination treatment suppressed the expression of GLI family zinc finger 1 (Gli1), which regulates the cell cycle, apoptosis, and proliferation, in the A549 cell line [56]. However, in drug resistance, the levels of ATP-binding cassette (ABC) transporters (ABCB1, ABCC10, and ABCC12), multidrug resistance 1 (MDR1), and multidrug resistance-associated protein 7 (MRP7), important factors in anticancer-drug resistance, were increased by NaB treatment in the A549 cell line, implying that MDR1 substrates (steroids, vinca alkaloids, and anthracyclines) may be less effectively regulated by NaB treatment [57]. Therefore, although the results of in vivo and in vitro studies have shown that SCFAs clearly suppress cell growth and metastasis of LC, SCFA treatment may need to be considered in lung cancer therapy to overcome drug resistance.

## Breast Cancer (BC)

**Fig. 2:**
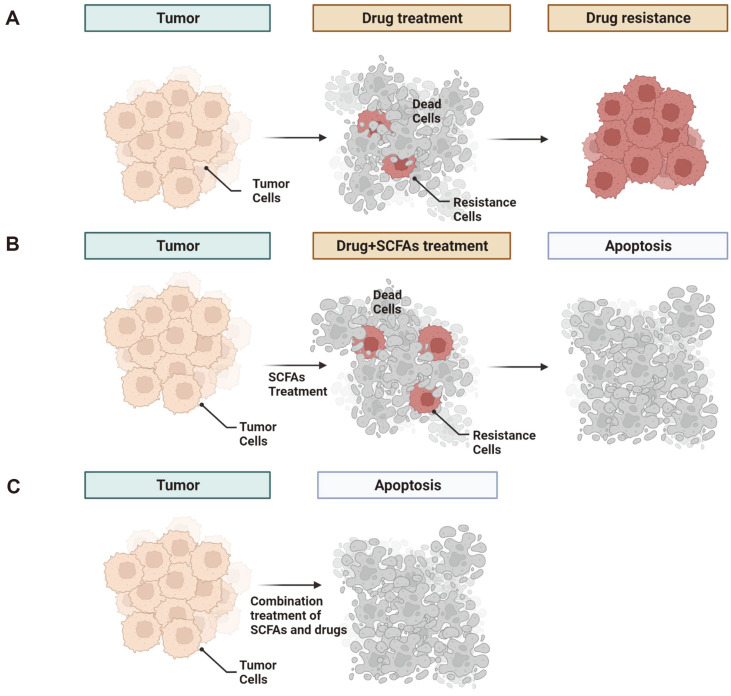
The anticancer roles played by SCFAs. Gut microbe-derived SCFAs induced cell apoptosis and reduced anticancer drug resistance, such as that associated with anticancer drug chemotherapy (**A** and **B**). SCFAs enhanced the synergistic effects of anticancer drugs (**C**).

BC is a cancer that largely affects females and is associated with high mortality worldwide. BC is classified into the luminal A/B, HER2-positive, and triple-negative types [58-60]. For these types of BC, an estrogen receptor (ER)-alpha antagonist has been widely used for the treatment of ER alpha-positive BC. To increase the efficacy of ER-alpha antagonists, treatment with NaB and NaP was analyzed, and the results showed that ER-alpha expression was reduced in MCF-7 and T47D cell lines and induced apoptosis [61]. Additionally, treatment with NaB and NaP induced cell cycle arrest in the G1 phase, as determined via flow cytometry analysis [62]. In addition, NaB treatment reduced superoxide dismutase (SOD), H_2_O_2_, and nitric oxide (NO) levels, and a high concentration of NaB affected DNA damage in the MCF-7 cell line [63]. TNBC cells do not express the ER, progesterone receptor (PR), or HER-2 receptor and present with distinct clinical features (high invasiveness, poor prognosis, and high metastasis) [64]. A standardized TNBC treatment has not yet been established [65]. In the MB-23-l TNBC cell line, combination treatment with sulforaphane (SFN), NaB, and genistein (GE; a DNMT inhibitor) resulted in G2/M-phase arrest. This combination treatment downregulated the expression of epigenetic modifiers such as DNMTs, HDACs, enhancer of zeste 2 Polycomb repressive complex 2 subunit (EZH2), SUV39H1, P300, PCAF and CBP genes, implying that this combination therapy and the use of dietary phytochemicals may be effective treatments for TNBC [66]. In engineering NaB treatments, gefitinib-loaded cellulose acetate butyrate nanoparticles (Gnb-NPs) formulated with chitosan hydrogel (Gnb-NPs-hydrogel) led to cytotoxic effects in the 4 T1 BC cell line in vitro and in vivo [67]. Therefore, to increase the drug efficacy of BC treatment, including TNBC treatment, SCFAs are expected to become important alternatives for synergistic anticancer effects and reduction of drug resistance (Fig. 2).

## Bladder Cancer (BA)

BA affects urinary bladder tissue and presents with symptoms such as blood in urine and pain with urination [68, 69]. BA is typically diagnosed in older people and is caused by immune dysregulation-related tumorigenicity induced by age-associated metabolic alterations in the bladder microbiome [70]. The administration of *B. pullicaecorum* to mice increased SCFA-related gene expression in mouse bladder urothelial cells, and an in vitro analysis showed that treatment with NaB induced apoptosis via an increase in FasL protein levels in the HT1376 bladder cancer cell line [71]. In addition, in T24 and 5637 bladder cancer cell lines, NaB treatment induced apoptosis and reduced the cell migration rate. EMT markers such as E-cadherin, N-cadherin, Vimentin and Snail were markedly changed by NaB treatment. Furthermore, miR-139-5p upregulation via NaB treatment led to decreased expression of B-lymphoma Mo-MLV insertion region 1-homolog (Bmi-1); subsequently, AMPK/mTOR pathway-related autophagy and excess ROS production led to apoptosis [72]. With a synergistic anticancer effect in BA, combination treatment with NaB and cisplatin increased the G1-phase arrest and apoptosis rates by regulating the expression of p21, p27, TNFRSF1A-associated via death domain (TRADD), and procaspase-2 [73]. Thus, gut microbe-derived SCFAs such as NaB are recognized as attractive anticancer drugs for BA treatment.

## Other Cancers

In hepatocellular carcinoma (HCC), NaB inhibits aerobic glycolysis by modulating c-myc signaling-related hexokinase 2 (HK2) expression. Sorafenib-induced upregulation of glycolysis was reduced by NaB treatment in vitro (in the LM3 and Bel-7402 HCC cell lines) and in vivo; hence, the anticancer effect of sorafenib was enhanced by NaB treatment [74]. In addition, NaB enhanced the effectiveness of gemcitabine in BxPC-3 and PANC-1 pancreatic ductal adenocarcinoma (PDAC) cancer cell lines. In an in vivo study, the group receiving a combination treatment consisting of gemcitabine and NaB showed a reduction in cancer-associated stromatogenesis and maintained intestinal mucosa integrity compared with the effect of gemcitabine treatment alone. Furthermore, in a fecal microbiota population, combination treatment with NaB and gemcitabine in a PDAC mouse model decreased the proportion of proinflammatory microbes [75]. Furthermore, the gut microbiota composition in acute myeloid leukemia (AML) patients and a mouse model showed a reduction in composition diversity. In particular, populations of butyrate-producing gut microbiota, such as *Faecalibacterium*, were significantly decreased in AML patients. The aforementioned AML mouse model showed intestinal barrier damage and lipopolysaccharide (LPS) leakage. However, butyrate treatment restored the intestinal barrier and reduced LPS leakage in AML model mice [76].

## Conclusion and Outlook

**Fig. 3:**
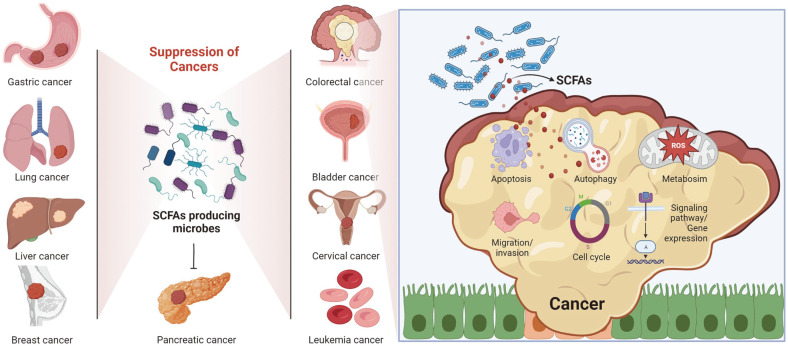
Anticancer effects of SCFAs in several types of cancers. SCFA-producing gut microbes and SCFAs, mainly butyrate, derived from the gut microbiota were closely associated with the suppression of cancer cell growth via their regulation of apoptosis, autophagy, metabolism, EMT process, the cell cycle, signaling pathways and onco-/tumor suppressor genes in several types of cancers.

Gut microbiota-derived SCFAs suppress cell proliferation and metastasis in several types of cancers, mainly CRC. Among these SCFAs, butyrate critically regulated apoptosis, autophagy, cell cycle arrest, migration/invasion, and cancer metabolic pathways by regulating the expression of cancer-related genes, signaling pathways, and translational modifications both in vitro and in vivo (Fig. 3). Combination treatment with butyrate and anticancer drugs enhanced drug efficacy and decreased the side effects associated with cancer treatment. Additionally, drug resistance to anticancer drugs was reduced by cotreatment with butyrate.

Together, with many advantages for cancer treatment, screening of several SCFA-producing gut microbes is expected to become an attractive approach to the development of anticancer therapeutic drugs. Moreover, for the treatment of rare cancers, SCFAs or SCFA-producing gut microbes are expected to become important alternatives, and SCFA engineering to develop cancer targeted-delivery systems will be continuously needed for an increase in drug efficacy. However, to use the gut microbiota or metabolites for cancer treatment, quality control (QC) of microbiota or metabolites, the determination of safe doses and other assessments are required during the development of gut microbiota-related cancer therapy.
